# Evolution of the Mammalian Ear: An Evolvability Hypothesis

**DOI:** 10.1007/s11692-020-09502-0

**Published:** 2020-05-27

**Authors:** Anne Le Maître, Nicole D. S. Grunstra, Cathrin Pfaff, Philipp Mitteroecker

**Affiliations:** 1 Department of Evolutionary Biology, University of Vienna, Althanstrasse 14, 1090 Vienna, Austria; 2 Department of Palaeontology, University of Vienna, Vienna, Austria; 3 PALEVOPRIM - UMR 7262CNRS INEE, Université de Poitiers, Poitiers, France; 4 KLI Institute for Evolution and Cognition Research, Klosterneuburg, Austria; 5 Mammal Collection, Natural History Museum Vienna, Vienna, Austria

**Keywords:** Inner ear, Middle ear, Mammals, Evolvability, Adaptation, Developmental instability

## Abstract

Encapsulated within the temporal bone and comprising the smallest elements of the vertebrate skeleton, the ear is key to multiple senses: balance, posture control, gaze stabilization, and hearing. The transformation of the primary jaw joint into the mammalian ear ossicles is one of the most iconic transitions in vertebrate evolution, but the drivers of this complex evolutionary trajectory are not fully understood. We propose a novel hypothesis: The incorporation of the bones of the primary jaw joint into the middle ear has considerably increased the genetic, regulatory, and developmental complexity of the mammalian ear. This increase in the number of genetic and developmental factors may, in turn, have increased the evolutionary degrees of freedom for independent adaptations of the different functional ear units. The simpler ear anatomy in birds and reptiles may be less susceptible to developmental instabilities and disorders than in mammals but also more constrained in its evolution. Despite the tight spatial entanglement of functional ear components, the increased “evolvability” of the mammalian ear may have contributed to the evolutionary success and adaptive diversification of mammals in the vast diversity of ecological and behavioral niches observable today. A brief literature review revealed supporting evidence for this hypothesis.

The vertebrate ear is a remarkable structure. Tightly encapsulated within the densest bone, the temporal bone, it comprises the smallest elements of the vertebrate skeleton (auditory ossicles) and gives rise to several different senses: the vestibular system with its semicircular canals enables balance, posture control, and gaze stabilization; the auditory system, including the cochlea and the ossicles in the middle ear, enables hearing. Nowhere else in the vertebrate skeleton are different functional units so close together and jointly embedded in its skeletal environment. The spatial and developmental entanglement closely integrates the variation of ear components and also links them to other regions of the cranium, especially the cranial base and the jaws (e.g., Luo 2011; Luo et al. 2017; Le Maître 2019). This is reflected by the observation that most of the human congenital malformations of the ear also affect other regions of the head (Wilkie and Morriss-Kay 2001; Kösling et al. 2009).

Even the growth pattern of the ear deviates considerably from that of the remaining skeleton: in humans and other mammals, the labyrinth achieves its final size already prenatally and the ossicles at very early postnatal stages (Anson and Cauldwell 1941; Roberto 1978; Eby and Nadol 1986; Yokoyama et al. 1999; Mennecart and Costeur 2016). This early cessation of growth challenges evolutionary change in the otic region because perinatal and postnatal development substantially contributes to cranial differences between many mammals otherwise (e.g., Garcia-Perea 1996; Cobb and O'Higgins 2004; Mitteroecker et al. 2004; Neubauer et al. 2010; Cassini et al. 2012; Singleton 2012).

All this makes it puzzling how mammals, as a predominantly nocturnal radiation reliant on hearing, were able to occupy such a vast diversity of niches in the aquatic, terrestrial, subterranean, and aerial realms that require an amazing disparity in locomotion, posture, and hearing abilities. How could the different, tightly connected parts of the ear adapt independently to these diverse functional and environmental regimes?

For example, even though birds comprise the more *diverse* clade in terms of recognized species numbers, mammals are much more *disparate* morphologically, behaviorally, and ecologically. Placental mammals alone span eight orders of magnitude in body size (compared to less than five orders of magnitude in birds) and occupy a wide diversity of niches, which is reflected in disparate morphologies and body plans associated with an impressive range of dietary strategies and modes of locomotion. The mammalian middle ear ossicles vary highly in shape, and different functional ear morphologies evolved as adaptations to low- or high-frequency hearing (including echolocation) and hearing in aquatic or subterranean life (e.g., Fleischer 1978; Mason 2013; Koyabu et al. 2017). Inner ear morphology, especially the shape of the semicircular canals, is closely linked to locomotor behavior and posture among mammals (e.g., Spoor and Zonneveld 1998; Spoor et al. 2007; Billet et al. 2012; Malinzak et al. 2012; Berlin et al. 2013; Le Maître et al. 2017; Pfaff et al. 2015, 2017). A comparison of older, more inclusive clades of birds and mammals does not alter the relative difference in disparity between the groups. Although Dinosauria exhibit more ecological, locomotor, and morphological disparity than birds alone, it nonetheless does not match the disparity observed in crown mammals, which includes fully aquatic and fossorial lifestyles as well as ultrasonic hearing, none of which are known to have evolved in Dinosauria.

Also the early evolutionary history of the vertebrate ear stands out from that of all other skeletal elements: Despite its functional and structural homology across all vertebrates, the ear is composed of different bones in mammals, birds, and reptiles. The transformation of the primary jaw joint into the middle ear ossicles in mammals is one of the most iconic transitions in vertebrate evolution, evidenced both by embryological and fossil data (Allin 1975; Maier 1990; Martin and Luo 2005; Rich et al. 2005; Meng et al. 2011; Mao et al. 2019). In non-mammalian amniotes, the lower jaw is composed of a tooth-bearing dentary and several post-dentary bones, including the angular and the articular. The latter forms the jaw joint with the quadrate, a bone of the cranium, behind which a single auditory ossicle, the columella auris, transmits the sound. In mammals, by contrast, the middle ear comprises multiple ossicles (malleus, incus, stapes) and one ectotympanic bone, supporting the tympanic membrane, all of which are separate from the jaw in extant mammals. Whereas the mammalian stapes is homologous to the single ossicle of non-mammalian tetrapods, the malleus and incus are homologous to the articular and quadrate bones, forming the primary jaw joint in non-mammalian jawed vertebrates, and the ectotympanic bone is homologous to the angular (Reichert 1837; Maier 1990). This evolutionary change was accomplished by several transformations of the respective hearing bones, which occurred multiple times independently (Rich et al. 2005; Martin and Luo 2005; Wang et al. 2019) and involved heterochrony and altered gene patterning in early mammalian embryogenesis (Luo 2007; Oka et al. 2007). However, the selective drivers of this complex evolutionary trajectory are not fully understood. It has been proposed that selection was initially not for hearing, but rather for mastication (e.g., Köppl and Manley 2018; Wang et al. 2019; Schultz 2020). Mao et al. (2019) suggested that the gradual incorporation of jaw joint bones into the middle ear of mammals eventually led to a decoupling of hearing and chewing modules, which may have enhanced their potential for independent adaptation to different selection regimes.

We propose here that this substantial evolutionary change of mammalian ear anatomy has—in addition to any direct enhancement and mutual decoupling of mastication and hearing—also increased the *evolvability* (capacity for adaptive evolution) of the ear and its associated sensory functions. The incorporation of the bones of the primary jaw joint into the ear has considerably increased the genetic, regulatory, and developmental complexity of the mammalian ear. For example, the mammalian middle ear derives from both the first and second pharyngeal arches, whereas in other amniotes only the second pharyngeal arch contributes to the ear bones (Sienknecht 2013; Anthwal and Thompson 2016). This increase in the number of genetic and developmental factors may, in turn, have increased the evolutionary degrees of freedom for an independent adaptation of the different functional units of the ear: the number of genetic and developmental “knobs” for natural selection to turn. This increased evolvability of the ear and its sensory functions may have contributed to the adaptive diversification of mammals and thus conferred a long-term fitness advantage to the mammalian clade.

Key to evolvability is the organism’s ability to generate heritable phenotypic variation along the encountered selective gradients (Kirschner and Gerhart 1998; Hansen and Houle 2008; Hendrikse et al. 2007; Melo et al. 2016). For a set of traits that serve different functions and thus experience different selective pressures, evolvability increases if the traits can vary independently; only then can the traits evolve independently and successfully respond to different selection (variational and evolutionary “modularity”; Wagner and Altenberg 1996; Wagner et al. 2007; Mitteroecker 2009). At the same time, traits that are functionally related should also vary in a coordinated way in order to evolve together without impairing their joint function. This congruence between functional relationships among traits and their pattern of (co)variation—as determined by the underlying genetic and developmental structure—reflects the key requirement for evolvability (Riedl 1978). But theoretical models have shown that independent variation and evolution of traits do not necessarily require completely independent genetic control (i.e., a modular genotype–phenotype map; Mitteroecker 2009; Pavličev and Hansen 2011; Pavličev and Wagner 2012). Instead, multiple pleiotropic genes with (partly opposite) effects on both traits can cancel and induce uncorrelated genetic variation. In fact, the greater the number of pleiotropic genes that affect a set of traits, the easier can the genotype–phenotype map adapt to the functional relationships among the traits. Compared to non-mammalian amniotes, the increased complexity in mammalian ear development involves a greater number of genetic and regulatory factors, cell migration patterns, and developmental interactions. The resulting increase in developmental variability likely translates into increased morphological variation that allows different functional units of the ear to adapt independently to different selection pressures.

It is disputed among evolutionary biologists—and target of numerous theoretical and computational research programs—as to whether the genetic-developmental structure can itself evolve by natural selection in order to increase the organism’s evolvability (e.g., Altenberg 1995; Jones et al. 2007; Wagner 2005; Wagner et al. 2007; Pigliucci 2008; Mayer and Hansen 2017; Payne and Wagner 2018; Mitteroecker et al. 2020). The mammalian ear is an important example here. Early evolutionary transformation of the cynodont ear presumably was driven by selection for mastication and later also for hearing (Köppl and Manley 2018). The anatomical decoupling of the mandible and the ear in the transition from Mesozoic mammaliaforms to the modern mammals has reduced indirect selective pressure on ear structures resulting from mastication, which in turn has facilitated further adaptation of hearing (Luo 2011; Mao et al. 2019; Schultz 2020). The incorporation of anatomical elements in the ear also increased the number of genetic and regulatory factors involved and thus the degrees of freedom for evolutionary adaptation. But this evolvability presumably evolved as a byproduct; it was not individual-level selection for evolvability, but for mastication and hearing that initiated the transition to the modern mammalian middle ear. Nonetheless, the evolutionary flexibility of the mammalian ear enabled the diversification of mammalian ear anatomy and, thus, conferred the entire mammalian clade a fitness advantage by helping mammals to occupy their wide diversity of niches as observed today.

As for most evolutionary hypotheses, our “evolvability hypothesis of the mammalian ear” cannot be evaluated directly, but it allows for a number of predictions that can be tested. For instance, we predict reduced integration (increased modularity) and a higher multivariate dimensionality of ear shape variation in mammals as compared with non-mammalian clades. As a result, we expect evolutionary rates and disparity of ear morphology to be greater in mammals than in non-mammalian clades of similar age. This includes a greater potential for evolutionary novelties (emergence of new anatomical structures; Peterson and Müller 2013) and for repeated or convergent evolution to arise in the mammalian clade. As a further result, we expect ear morphology to correlate more tightly with ecological, behavioral, and auditory variables in mammals as compared with non-mammalian clades. Finally, we predict that the increase in developmental and variational complexity of the mammalian ear has a price: it may make the ear more susceptible to developmental instabilities and disorders. Mammals may thus show a greater degree of fluctuating asymmetry in ear shape than other clades and perhaps also a higher incidence of otological disorders.

To our knowledge, no quantitative studies comparing ear shape variation between mammals and non-mammalian amniotes have been conducted so far. But our predictions are supported by a range of morphological studies in different vertebrate species. For example, semicircular canal shape tightly correlates with locomotor behavior, posture, and agility in mammals (e.g., Spoor and Zonneveld 1998; Spoor et al. 2007; Billet et al. 2012; Malinzak et al. 2012; Berlin et al. 2013; Le Maître et al. 2017; Pfaff et al. 2015, 2017), whereas shape differences in the avian vestibular system seem to be due mainly to variation in body mass and brain size rather than flight behavior, even though coordination abilities strongly depend on vestibular stimuli and their reflexes (Benson et al. 2017; Sipla 2007). In lizards, semicircular canal shape was found to correlate with some microhabitats, but this association presumably results from allometry and spatial constraints of the skull (Dickson et al. 2017; Vasilopoulou-Kampitsi 2019a, b). Both in reptiles and birds, the length of the cochlear duct of the inner ear correlates with hearing abilities (Walsh et al. 2009); however cochlear morphology, notably the length and number of coils, is much more diverse in mammals than in non-mammalian clades (Gleich and Manley 2000; Ekdale 2013, 2016), which is associated with the extensive variation in the ranges of hearing frequencies among mammals (Manley 2012).

As for the middle ear, five functional types have been distinguished in placental mammals (Fleischer 1978), all of them linked to specific environments and behaviors, representing multiple examples of convergent evolution. For instance, the “microtype” ossicular morphology is associated with high-frequency hearing and typical of various, phylogenetically diverse, small mammals (Rosowski 1992; Mason 2013), and the “freely-mobile” middle ear type is associated with low-frequency hearing and evolved independently in medium-sized terrestrial mammals and subterranean mammals (e.g., Mason 2013, 2016; Koyabu et al. 2017). Another example of convergent evolution includes exceptionally dense tympanic and periotic bones in several aquatic mammalian clades (Fleischer 1978; Ketten 1992). In non-mammalian clades, on the contrary, functional associations of middle ear shape seem to be less pronounced. For example, no correlation was found between habitat ecology and shape and size of the middle ear cavity in turtles (Foth et al. 2019). Also, the size of middle ear structures was linked to auditory abilities within each class of tetrapods, but this relationship is largely driven by variation in body size (Gridi-Papp and Narins 2009).

Increased evolvability is further evidenced by the number of mammalian novelties in the ear compared to other tetrapods. Only mammals have evolved a sophisticated outer ear with an auditory canal and a pinna, which, at least among therians (marsupials and placentals), varies extensively in size, shape, and degree of mobility, enhancing sound detection and localization (Webster 1966). Furthermore, early mammals co-opted the angular bone to form the ectotympanic bone, and they evolved two to three additional middle ear ossicles (Luo 2011; Meng et al. 2011; Han et al. 2017; Mao et al. 2019) at least three times independently over the course of their evolution: in monotremes, allotherians, and trechnotherians (Luo 2011; Wang et al. 2019). The organ of Corti with its electromotile outer hair cells is another crucial mammalian novelty that enabled to perceive high-frequency sounds, exploit a variety of new niches, and develop new vocalization patterns throughout mammalian evolution (Brownell et al. 1985; Dallos et al. 2008; Ashmore et al. 2010; Manley 2012). The therian cochlea lengthened to several times of that observed in birds and other tetrapods (Manley 2012); in placentals a high degree of cochlear coiling evolved multiple times independently (Ekdale 2013).

Clearly, systematic quantitative studies of the (co)variation of ear structures and their functional associations within different mammalian and non-mammalian clades of comparable age are necessary for effective comparisons of ear evolvability. But the existing literature already indicates a considerably greater capacity of adaptive evolution of the ear in the mammalian lineage as compared to other clades, which has resulted—according to our hypothesis—not only from the decoupling of ear and mandible but also from the increased developmental complexity in the mammalian ear.
